# “I’m Not a Therapist—I Am a Streamer”: An Interpretative Phenomenological Analysis of Twitch Partners’ Experience of Mental Health Within Their Communities

**DOI:** 10.3390/bs16071161

**Published:** 2026-07-10

**Authors:** Daniel Lodge, Anastasia Rousaki, Rory Colman, Robyn Mooney, Dean Fido

**Affiliations:** 1 School of Science, College of Science and Engineering, University of Derby, Derby DE22 1GB, UK; daniellodge.edu@gmail.com (D.L.); r.colman@derby.ac.uk (R.C.); 2 Division of Nursing, Midwifery and Social Work, School of Health Sciences, University of Manchester, Manchester M13 9PL, UK; anastasia.rousaki@manchester.ac.uk; 3 Department of Clinical Psychological Science, Maastricht University, 6211 LK Maastricht, The Netherlands; robyn.mooney@maastrichtuniversity.nl

**Keywords:** Twitch, mental health, content creation, digital labour

## Abstract

Mental health remains a global concern for which the burden of care frequently falls onto non-clinical platforms. This study employed Interpretative Phenomenological Analysis to explore how *Twitch.tv* is unexpectedly being used to curate communities wherein mental health is discussed and supported. Using semi-structured interviews with six Twitch Partners, we generated three superordinate themes: 1. Positive Experiences of Streaming, 2. Negative Psycho-social Sense-making in Relation to Streaming, and 3. Emotional Dilemmas Regarding Stream-related Payments. Twitch Partners face challenges around balancing pressures of generating and maintaining communities, whilst addressing individual relationships and the needs of members within. This paper documents strategies that Twitch Partners employ to protect their own mental wellbeing whilst supporting their communities, and notes how monetised content can impact such strategies and generate additional stress. These findings highlight the need to explore and evaluate platform support for streamer wellbeing, including signposting, moderation resources, mental health guidance, and training.

## 1. Introduction

With the rising prevalence of anxiety, depression, and loneliness impacting societies, mental health remains a pressing global concern (World Health Organization, 2025). Although public awareness of mental health has grown, most individuals experiencing mental health issues do not receive adequate treatment (Thornicroft et al., 2016). For example, around 8 million people struggle to access timely mental health services in England through the National Health Service (Campbell, 2021), and around 28 million people lack access to mental health treatment in the United States (The State of Mental Health in America, 2023). This treatment gap is widely recognised, and further hindered by systemic barriers, such as underfunding and ineffective policies, in addition to financial and logistical constraints (Campbell, 2021). Consequently, the burden of care falls onto non-clinical systems (Thornicroft et al., 2016), such as digital platforms (e.g., discussion forums) wherein one seeks validation and solidarity through sharing experiences and engaging in open and non-stigmatising discussion (Gandhi et al., 2021; Gonsalves et al., 2023; Griffith & Stein, 2021).

Among these platforms, live-streaming services, such as *Twitch*, *YouTube*, *TikTok*, and *Kick* have emerged as unexpected yet significant venues for mental health disclosures. Live-streaming services utilise integrated chat functions to facilitate real-time communication between streamers (those playing video games and/or conversing about topics of interest) and their (largely anonymous) audiences. Such audiences who can further engage by sending donations, playing interactive games, and joining communities hosted on third-party sites (Zhao et al., 2018). This sustained interactivity cultivates a sense of community and shared experience (Hamilton et al., 2014), blurring boundaries between entertainment, networking, and emotional labour (Hamilton et al., 2014; Sjöblom & Hamari, 2017). Though sometimes criticised for fostering negativity (de Wit et al., 2020), live-streaming services have become platforms for mental health discourse (Hilvert-Bruce et al., 2018), wherein streamers and their audiences can discuss mental health, disclose personal struggles, and seek support without fear of stigma (Robinson, 2019; Uttarapong et al., 2022).

Streaming communities have been linked to psychological well-being, including stress relief (C. Y. Chen & Chang, 2019), emotional regulation (Sjöblom & Hamari, 2017), life satisfaction (Kim & Kim, 2022), and belonging (C. C. Chen & Lin, 2018). Indeed, viewers report watching streams as a coping mechanism that offers distraction and emotional support (de Wit et al., 2020). However, despite individuals strengthening their social capital by actively engaging in digital communities (Wolff & Shen, 2024), viewers can develop parasocial relationships with streamers (Speed et al., 2023); leading to unrealistic expectations and an over-reliance on digital interactions (Lim et al., 2020).

On the other side of the camera, streamers also report benefits, such as reduced loneliness and stress, and provision of a structured routine (C. C. Chen & Lin, 2018; Sjöblom & Hamari, 2017). However, there is growing recognition of the emotional burden placed on content creators (Wallace et al., 2026). Streamers experience logistical pressures related to technological difficulties (Woodcock & Johnson, 2019) and community management, such as always feeling online and being expected to cultivate supportive environments (Speed et al., 2023; de Wit et al., 2020). They also report experiencing unsolicited disclosures of trauma, as well as emotional exhaustion from moderating sensitive and emotionally charged topics (Uttarapong et al., 2022). Using grounded theory, Gandhi et al. (2021) highlights how mental health discussions within streams are spontaneous, with unprepared streamers playing an unexpected role in enabling emotional connections. Though conversations help normalise mental health discourse (thereby reducing stigma), some viewers prefer to use streaming as entertainment, forcing streamers to manage their online identities by balancing empathy and authenticity with maintaining audience expectation and engagement (Woodcock & Johnson, 2019). This point is underlined by reports outlining extant structures as being detrimental to the mental health of content creators (Powell, 2022).

Compounding this is a view of streaming platforms as sites of “playbour” where play blends with monetised labour, sustaining a culture of inaction toward harassment and marginalisation, particularly affecting women and minority groups (Catá, 2019). This occurs because monetisation structures (i.e., subscriptions, donations) encourage streamers to remain constantly available and tolerate contrasting audience behaviours to maintain engagement and financial support. As a result, streamers endure or normalise toxic interactions, including sexism, racism, and other forms of abuse, rather than risk alienating paying audiences (Catá, 2019). Indeed, streaming can be viewed through a capitalist lens wherein cultivating relationships, maintaining a persona, and delivering an ongoing performance of authenticity is framed as leisure but operates as intensive, precarious labour. Monetisation makes the boundaries between work and play more porous, and while the activity is popularly imagined as “*playing games for a living*”, it in fact demands specialised labour (Woodcock & Johnson, 2019).

### Present Study

Despite a wealth of social scientists and charity sectors providing education and support for engaging in online environments, there remains a scarcity of peer-reviewed understanding of streamers’ wellbeing. This study examines how Twitch Partners make sense of their role when entertainment, community care, mental health disclosure, and monetised labour overlap. The theoretical contribution of this study lies in showing how emotional labour and monetised digital labour are experienced as interconnected role dilemmas. Participants experienced streaming as simultaneously a source of self-care, a form of community care, a creative performance, and a monetised activity. Such conceptualisations produced tensions around responsibility, authenticity, boundaries, and payment.

## 2. Method

### 2.1. Theoretical Approach

Interpretative Phenomenological Analysis (IPA) explicated how individuals made sense of their experiences through detailed, first-person accounts. IPA is grounded in idiographic and hermeneutic phenomenology, prioritising meaning-making at the individual level rather than seeking generalisable causes. IPA aligns with phenomenologists such as Merleau-Ponty, who argue that experience is situated, embodied, and relational. As such, IPA acknowledges the interpretative nature of phenomenological inquiry, emphasising that individuals engage with the world from a specific context. This focus is particularly suited to the exploration of personal and psychological meanings (Smith, 1996).

IPA is concerned with how individuals relate to the world through meaning-making and operates on key assumptions: understanding the world requires understanding experience, individuals are embedded in linguistic, relational, cultural, and physical contexts, and research must adopt idiographic approaches to capture personal meaning. Researchers elicit detailed first-person accounts, highlighting the subjective nature of experience. Its commitment to idiography ensures a focus on the particular, enabling a nuanced exploration of lived experiences within broader contextual frameworks (Larkin & Thompson, 2011). It is particularly useful for capturing reflective experiences in psychology, health psychology, and related fields (Larkin et al., 2006; Smith, 1996) and is well-suited for exploring mental health (Larkin & Thompson, 2011).

### 2.2. Participants

In the context of IPA samples typically being small and homogeneous (Cena et al., 2024), six Twitch streamers (*n* = 5 men, *n* = 1 woman) were recruited from the UK, Norway, and the USA between September 2022 and February 2023; allowing for depth of data, whilst maintaining logistical feasibility of recruiting such niche demographic. Participants were recruited through snowballing and were over 18 years of age, fluent in English, and of Twitch partner status (i.e., having maintained an average of 75 concurrent viewers within a 30-day period and showing growth potential). Given the nicheness of streaming, we worked with each participant prior to their interviews to help them self-selected pseudonyms to maintain anonymity, including giving guidance on avoiding location-specific wording and phrases that encompassed common terms/points of discussion within their online communities.

In line with IPA’s idiographic orientation, the study prioritised interpretative depth instead of analytic saturation. The sample was therefore judged sufficient not because it exhausted all possible experiences of Twitch streamers, but because it enabled detailed case-by-case analysis of a small, relatively homogeneous and hard-to-reach group. This allowed us to explicate how participants made sense of their roles within this complex context. The gender imbalance and cross-national composition of the sample have also potentially shaped the interpretative scope of the study. The predominantly male sample limits our ability to examine how emotional labour may be gendered. Similarly, participants’ different national contexts may have shaped their experiences. The findings should therefore be read as a situated idiographic account, rather than as representative of Twitch streamers more broadly.

### 2.3. Analytical Procedure

Data were analysed in line with Smith et al. (1999). The process began with data immersion; repeatedly reading transcripts while maintaining reflexive notes, followed by line-by-line coding; identifying key objects of concern (what mattered to participants) and experiential claims (how they interpreted experiences). Themes for each case were explicated before case-to-case analysis explored similarities/differences across participants. Next, we developed an interpretative account, linking themes to psychological/socio-cultural concepts, and constructed a structured representation to organise findings coherently. Finally, we presented (sub)themes alongside quotes and interpretations. We ensured rigour by maintaining a level of reflexivity, peer validation, and transparency in data tracing. IPA remained iterative, requiring multiple cycles of engagement with the data (Cena et al., 2024).

The data were analysed in line with IPA principles, prioritising idiographic depth before moving to cross-case interpretation. The first phase of analysis involved one researcher reading the transcripts repeatedly and developing an initial set of themes. A second researcher then independently familiarised themselves with the dataset, revisited the relevant analytic sections, and developed further exploratory notes on participants’ accounts. Initial experiential claims were refined for each case, focusing on key emotional meanings, and participants’ sense-making around streaming, community responsibility, mental health disclosure, negative feedback, and monetised support.

Analysis proceeded case-by-case before moving to cross-case comparison. Case-level themes were generated for each participant and then compared across the sample, with related case-level themes clustered into the final superordinate and subordinate themes. The two analytic phases were then brought together, allowing the researchers to refine the thematic structure, consolidate overlapping interpretations. Peer validation involved reviewing the developing theme structure and refining claims. This process supported a clearer distinction between participants’ own meaning-making and the researchers’ interpretative extensions.

It is noteworthy that following the double hermeneutics approach, we adopted a critical psychology interpretation of the dataset, focusing on how labour and care are experienced in the dataset based on the socio-economic context and platform dynamics of streaming (Rousaki, 2026).

### 2.4. Materials and Procedure

The study was approved by a College Research Ethics Committee at the University of Derby (ETH2122-4608). After responding to an e-mail invite, participants provided informed consent and information about their age and sex via a Qualtrics survey. One-to-one semi-structured interviews were arranged and conducted/recorded via Microsoft Teams by DL (interview schedule here: https://osf.io/hgxuz/?view_only=6eced043e2ea4eb59c3a5a6164292d27, accessed on 6 July 2026), each lasting 45–60 min. Participants were thanked and debriefed; giving opportunity to discuss the process with each participant to ensure that they were not in distress, and had access to any signposting they required. Interviews were automatically transcribed using the Microsoft Teams transcription feature, before being manually checked for accuracy thereafter. Recordings and transcripts were stored on an encrypted OneDrive, accessible only via the research team. Recordings were retained until all transcripts had been analysed, with transcripts retained until the point of publication.

During the manuscript drafting process, the research team were mindful of deductive disclosure risks. Despite it not being required, we operated an analytic approach wherein quotes deemed important to our narrative that risked reducing anonymity (e.g., country- or game-specific narratives, or those citing well-known events), would have been re-worded whilst maintaining meaning (as verified by participants). Otherwise, quotes were not confirmed with participants, but they were given opportunity to remove any data they subsequently wished to after participating.

### 2.5. Reflexivity

Since IPA emphasises the researcher’s interpretative role, we remain mindful of its epistemological and ethical implications. Our reflections were influenced by several factors. First, our background in digital technologies, mental health, and online communities provided substantial and specialised insight into the affordances and challenges of online spaces. While helping us engage with accounts, we bracketed assumptions to ground interpretations in lived experiences rather than preconceived ideas. Second, the relational nature of streaming added complexity to data analysis. DL and DF have experience with streaming live content and community formation, and AR, RC, and RM were aware of negative experiences faced by streamers and had familiarity with concepts regarding digital labour. During analysis, DL and AR used reflexive notes to document thoughts/emotional responses. Finally, our findings reflect a specific technological context. As streaming platforms evolve, so do the socio-emotional dynamics of content creation, which affects our interpretations.

Our reflexive position was informed by critical psychology and by IPA’s double hermeneutic, whereby participants make sense of their experiences and researchers, in turn, interpret that meaning-making. This orientation enabled us to read participants’ accounts as both embodied as well as socially situated, particularly in relation to digital labour and platform-mediated community life. Yet, we recognise that this critical orientation could lead us to interpret accounts through broader theoretical frameworks.

## 3. Results

In line with IPA’s idiographic orientation, themes are presented as patterned meanings across cases rather than claims of representativeness. Not all participants expressed each theme in the same way or with equal intensity. More specifically, we explicated three superordinate themes: [1] *Positive Experiences of Streaming*, [2] *Negative Psycho-social Sense-making in Relation to Streaming*, and [3] *Emotional Dilemmas Regarding Stream-related Payments*. Table 1 therefore indicates where each theme was evidenced across participants.

### 3.1. The Positive Experiences of Streaming

#### 3.1.1. Streaming as a Coping Mechanism to Psycho-Social Challenges

Participants appear to interpret streaming as more than a leisurely activity, serving as a means of self-care, escapism, and self-fulfilment. Indeed, participants describe their experience of streaming as allowing them to “*decompress*” and pursue happiness and fulfilment, contrasting it with other demanding or challenging areas of life. In many ways, their sense-making of streaming frames it as an activity that provides a structured refuge from life’s difficulties:

***Excerpt 1, Jared:** I just did it as like to kind of kill a bit of time for myself and it was like a destress for myself because my day-to-day job is quite stressful, high pace, it was a way for me to kind of relax and sort of decompress my life *[lines omitted]* it’s about escaping reality, let’s say, it’s about escaping my everyday life and taking this portion of my life and using that to destress, decompress.*

Jared’s sense-making of the benefits of streaming is characterised by a strong juxtaposition to the high-paced and demanding nature of his job. His narrative frames streaming as more than just a hobby, and by emphasising words such as “*relax*”, “*decompress*”, and “*destress*” he communicates streaming as a form of self-care.

Jared’s account may suggest that streaming serves as a way of managing stress and emotionally unwinding after work. Jared’s interpretation of the benefits of streaming implies a soothing effect on his overall levels of stress, and his sense-making is further reinforced through escapist analogies and repeated references to decompression. Jared’s phrase “taking this portion of my life” suggests that streaming is experienced as a distinct part of his everyday life, positioned in contrast to the pressures of his job. This could be interpreted as an attempt to create some separation from work-related demands, through a space that feels more controllable. The repetition of “*destress*” and “*decompress*” further highlight both the relief streaming provides. However, from a double hermeneutics standpoint, streaming as escapism is framed both positively but also to distance himself from what appears to be a substantial stressor (i.e., work stress). Despite its complexity, this was a common experience among streamers, as reflected in Peter’s account:

***Excerpt 2, Peter:** For me, it’s just about trying to find how I can be at my happiest in life and… and I figured that I do think this is the right path for me, so it’s something I’m really passionate about… *[lines omitted]* Of course, I can probably say that there’s some yeah, escapism from the… the hardships of life in general, so there’s… it’s a bit of everything, really.*

Peter’s account signals a personal investment in streaming; his phrases “*trying to find how I can be at my happiest*”, “*the right path for me*”, and “*I am really passionate about*” highlight that while experiencing significant happiness through streaming, his sense-making extends beyond positive emotions to a pursuit of meaning and self-fulfilment.

Peter also acknowledges an element of escapism, describing streaming as a source of relief from “*the hardships of life in general*”. Like Jared, Peter’s understanding of streaming is shaped by a strong contrast with the challenges of everyday life, positioning streaming as a means of emotional and psychological respite and a form of escapism. His final statement, “*it’s a bit of everything, really*” suggests that escapism and passion are intricately connected. In many ways, both Peter and Jared experience streaming as a means of decompression wherein their lived experiences highlight a separation between streaming and other aspects of life. Streaming might be experienced as a chosen activity, perhaps signifying agency, whereas other domains may feel more obligatory.

#### 3.1.2. The Joy and Responsibility of Facilitating Communities

Participants interpret streaming as a fundamentally relational practice centered on community-building and emotional support. Their interpretation of their identity is that of facilitators who create and maintain a cohesive, inclusive space, capable of collectively celebrating personal milestones and attending sensitively to the emotional complexities of their audience. Through repeated references to unity and care, participants conceptualise streaming as an emotionally responsive environment where interactions can influence their audience’s mental health and wellbeing:

***Excerpt 3, Stewart:** I have had people express, you know, come in with great news. You know, it’s like, hey, I just got the promotion. Or hey, I just, you know, I just checked my weight this morning. I’m down forty pounds from where I was six months ago. Like I’ve, we’ve and we get to celebrate in that moment with them and we share those moments as a community too. So like through the good and the bad, I want to make sure that we’re all together and we’re all feeling cohesive.*

Stewart’s interpretation of being a streamer reflects a sense of responsibility towards facilitating and creating a supportive and welcoming community. Examples of audience members sharing personal achievements (e.g., promotions, weight loss) demonstrate his perception of streaming as a reciprocal and communal domain. Perhaps Stewart feels that streaming is a meaningful opportunity for collective emotional support within a community.

Stewart’s identity as a streamer can be interpreted as being a facilitator through whom community members’ positive and negative messages are conveyed, allowing him to hold his community together. His repeated use of inclusive language (e.g., “*we get to celebrate in that moment with them*”) perhaps suggests that he interprets streaming as a privilege (highlighted by the phrase “*get to*”) and a collective endeavour. This communal experience is characterised by a sense of belonging, active engagement, connection, and support. Moreover, the phrase “*through the good and the bad*” indicates that Stewart views emotional engagement in streaming as continuous, not conditional. Perhaps, Stewart perceives his community as a space to maintain emotional connectivity across varying affective states. In many ways. Stewart perceives streaming as a space where real-life emotions and personal experiences intersect and interact. Similarly to Stewart, Jared’s sense-making was related to building and maintaining a community:

***Excerpt 4, Jared:** …but I also think for me personally it’s a big thing about community and the community that come to me for…. I don’t know their day-to-day lives…. I don’t know what’s going on in their personal life and for me knowing that I’ve made them laugh made them smile, made their day that slight bit better…*

Jared conceptualises his streaming as inherently tied to community support and well-being; perhaps feeling that streaming is an environment attentive to the emotions of his audience. Jared acknowledges his limited awareness of viewers’ individual circumstances (“*I don’t know their day to day lives*”) and, in many ways, highlights the contrast between the visible, public responses to his content and the emotional struggles that viewers may privately carry. Jared’s sense making of interactions suggest that they occur precisely at this intersection, emphasising the potential to positively affect viewers’ wellbeing, even in the absence of detailed knowledge about their lived experiences and day-to-day realities. In articulating his role as someone who can “*make someone smile*” or “*brighten their day*”, Jared potentially indicates an understanding of emotional impact as incremental but significant. Jared’s repeated emphasis on relationality and care is like Stewart’s, in that he appears conscious of the responsibility he holds toward community members, whose personal struggles, though largely invisible to him, still matter within the streaming community and domain. This understanding of streaming as being emotionally responsive, makes him recognise his role within the community as extending beyond an individualised performance. His sense-making suggests that he understands streaming as a role that involves a degree of informal care and emotional attentiveness toward viewers.

### 3.2. Negative Psycho-Social Sense-Making in Relation to Streaming

#### 3.2.1. Emotional Labour and a Sense of Responsibility

Participants also discussed negative psycho-social experiences of streaming. They discussed how audiences perceive them as being in a position of responsibility, with many viewers discussing their mental health in ways that felt overwhelming. Participants experienced a misplaced emotional labour, wherein they often bear the weight of the viewers’ mental health. They also discussed how such incidents negatively affected their own health; leading to a negotiation of their own identity, and a need to define boundaries around what their role as a streamer is. For example, Jared discussed implications of mental health disclosures and how it affects him:

***Excerpt 5, Jared:** And like I said, they dump it on you and it’s your then sort of responsibility as the content creator, either make them feel welcome or maybe you can’t do it. You can’t because of… I think the more you try, lug on yourself to look after them, it’s gonna be harder for you. It’s like anything the way I sort of see it is. The more people put their emotional states on me, it’s like a backpack. I’ll carry that around on me all day until it gets too heavy the number and I’ll get upset and I can’t deal with that for myself, I think.*

Jared’s account highlights the emotional labour associated with managing a community as a content creator; describing how his audience projects their emotional states and, in doing so, “*dumps*” responsibility onto him, which makes streaming an accumulative challenge. This fragmented experience presents a contradiction; Jared struggles with balancing audience expectations and his own emotional capacity: “*you can’t because of… I think the more you try…*”; highlighting the dilemmas he experiences when navigating the weight of responsibility while maintaining his own emotional well-being.

Jared metaphorically describes this burden as “*like a backpack”*. Such phrasing is perhaps a way of suggesting he makes sense of this emotional responsibility as something heavy and cumulative; eventually becoming overwhelming. In this sense, he sees a misplacement of responsibility within his role as a streamer. The cumulative effect is evident in his remarks: “*it’s going to be harder for you*” and “*it gets too heavy*.” His statement, “*I’ll carry that around on me all day until it gets too heavy*” suggests his role can result in feeling a shift from endurance to emotional exhaustion. He acknowledges a threshold beyond which the emotional weight becomes unmanageable. His final remark, “*I’ll get upset and I can’t deal with that for myself*” reveals how these demands might make it challenging to manage his own well-being. One can observe similar sentiments in Anna’s perspective:

***Excerpt 6, Anna:** …trauma dumping is bad for my mental health. Of course, I get to know unnecessary, in my opinion, unnecessary information about this person and that they could have not said everything. I understand that you can’t always say ‘Ohh yeah, I’m good’, Whatever, but like going in detail and things, is in my opinion, unnecessary. At least it’s like, I’m not a therapist. I am a streamer and entertainer and all that. I can’t really help with that stuff anyways.*

Anna’s identity as a streamer is at odds with the responsibility imposed on her. Similarly to Jared, who used the word “*dump*” she uses the words “*trauma dumping*”, potentially indicating that she feels a sense of misplaced emotional labour. Her repeated use of “*unnecessary*” could reflect efforts to make sense of the emotional strain being experienced and frustration with being expected to engage with others’ trauma, her feeling of being overwhelmed by the weight of these disclosures, and her belief that such expectations do not align with her role. This disconnect appears to add to her struggle.

The weight of receiving personal disclosures seems to affect Anna’s well-being, forcing her to reconcile her role with expectations placed upon her. This burden is neither one she agreed to nor one she feels equipped to bear. She attempts to define boundaries between what she believes her role should be and what it has become. More specifically, unlike Jared, who asserts that this type of responsibility is embedded within the role of the streamer, Anna seems to resist this notion (“*I am not a therapist, I am a streamer…I can’t really help with that stuff*”). Within this response, Anna’s sense making of her role is that she is not obligated to carry this emotional burden More specifically, her insistence that she is “not a therapist” can be interpreted as an attempt to re-establish the limits of her role and to distance herself from forms of emotional responsibility that she does not feel equipped to provide.

In many ways, Anna’s identity as a streamer appears to be at odds with the responsibility she experiences as being imposed on her, creating a strain on her mental health. While the present analysis does not support a full gender-based interpretation, expectations of care and within digital spaces are often gendered (Rousaki, 2026).

#### 3.2.2. Managing Negative Feedback

Participants experience streaming as intertwined with validation and rejection, and highlighted the unpredictable nature of audience interactions. They described the impact of external feedback on their emotional well-being, with positive recognition offering them a sense of affirmation, and negativity—whether through rejection of the content they produced or unsolicited hostility—creating feelings of dismissal and frustration. Their experiences note vulnerability characterising creative work, which entails emotional investment, effort, and exposure. Kevin frames audience feedback as a powerful force shaping his sense of worth:

***Excerpt 7, Kevin:** Picture yourself at five years old, colouring a picture for your mom and she puts it on the fridge. That’s a warm fuzzy feeling. Now let’s just say this is disgusting. She crumples it up and throws in the trash. You know, how does 5-year-old you feel right? Like, probably a lot more feeling at five than you would have as, you know, an adult. But the feeling is still the same so when the community is outwardly, like, negative about this creative project you’ve put together, it hurts like, you don’t, it’s not a positive feeling particularly, you know, and I think it does scale to how many hours you’ve put into this project.*

Kevin employs strong metaphors to make sense of both positive and negative interactions with his audience; his description of validation likens positive feedback to a child whose mother proudly displays their artwork. This metaphoric comparison could suggest that acknowledgement provides a sense of visibility and affirmation; showcasing the central role the audience holds in Kevin’s streaming experience.

However, these positive feelings exist in the same spectrum as the experience of rejection. The shift from the metaphor of the fridge to the metaphor of throwing something in the trash exemplifies the contradictory and binary nature of audience feedback, one moment affirming and fulfilling, the next abrupt and invalidating. His use of the word “*disgusting*” to describe the metaphorical act of a mother crumpling the painting could signal a substantially emotional interpretation, again due what appears to be to the audience’s vital role in how he experiences streaming. Kevin’s metaphor may suggest that negative audience feedback evokes feelings of rejection and invalidation. Perhaps the level of hurt experienced is directly proportionate to the effort he puts in; greater investment leads to greater emotional vulnerability.

While Kevin acknowledges that an adult might rationalise criticism differently, he argues that the emotional response remains largely unchanged. He recognises the enduring nature of feelings of rejection and validation. In many ways, he frames the audience as more than a group of viewers; Kevin might, perhaps, feel that the audience is a powerful external force capable of determining his emotional state. He experiences public scrutiny in a way that feels personal, as his creative work is met with unfiltered reactions.

Kevin appears to be personally and creatively invested, whereas the audience has the freedom to critique or dismiss without the same stakes. He faces rejection as a challenging experience, one that can be read as shaped by emotional history/pre-existing affordances, creative-albeit publicly visible-investment. His reflections could be interpreted as indicative of the challenges of meaning-making in digital creative work, where validation is external, unpredictable, and often volatile. Anna experiences similar feelings in relation to negative interactions as a streamer, but differently to Kevin, Anna manages this through the enforcement of boundaries:

***Excerpt 8, Anna:** I mean the type of negativity that can impact my mood is like someone coming in trolling or like, like hating immediately from first chat and uh, yeah. But I try my best to immediately ban them. Sometimes it’s kind of… I want to like know what’s going on, but like you should just like ban them and like move on honestly.*

Anna describes a distinct form of negativity that is unsolicited and disruptive. She does not bring up general criticism, but focuses on trolling and hostility that emerge from the first interaction. What makes this negativity striking is being subjected to its abruptness and lack of context (“*immediately, from the first chat*”). Anna acknowledges the impact of these encounters on her mood. Such interactions seem to contradict the positive communities and self-fulfilment described in Theme 1, with this juxtaposition further pronouncing the negative sentiments they cause to the participants.

To counteract this, Anna employs a firm approach through immediate banning, which could be interpreted as a commitment to maintaining and enforcing emotional boundaries. However, this creates internal conflict: “*Sometimes it’s kind of… I want to like know what’s going on*”. Perhaps, while she understands the importance of shutting down negativity, she also feels the need to understand its origin. Her concluding remark *(“You should just like ban them and move on honestly*”) reinforces the idea that detachment is her preferred response for self-protection. Her identity as a streamer could be interpreted as requiring continuous negotiation; staying engaged and receptive to an audience while being prepared to emotionally manage and practically shut down harmful interactions.

### 3.3. Emotional Dilemmas Regarding Stream-Related Payments

Streamers experience monetary donations as dilemmatic. Initially, they interpreted accepting monetary support as misaligned with their identity and social connections. Financial contributions made them feel awkward, uneasy, and resistant, as if accepting money was somehow wrong. However, over time, participants experienced what can be interpreted as a shift, experiencing transactions as forms of validation and community engagement rather than mere financial exchanges. This shift allowed them to reconcile their discomfort, recognising donations as an expected and legitimate expressions of support. A similar discomfort which is then resolved through feelings of legitimisation is described by Kevin:

***Excerpt 9, Kevin:** So, it’s been a struggle for me personally. If some random person on the street comes up and gives me $5, I would have a lot of questions. I likely would not take them on. That’s just the kind of person I am… Every stream, I you know, my community is advanced to a level where literally every stream is, you know, has monetary value assigned to it. But it’s still awkward for me. It is affirming if I’m being totally honest. There is this feeling in humanity where people will say whatever, but they mean it when they put their money behind it, right?*

Kevin’s account echoes of a sense of conflict around payment: although he describes monetary support as “affirming”, he also frames it as “awkward” and as a personal “struggle”, suggesting that being paid feels difficult and unnatural to him. To convey the novelty, and discomfort of this experience, he employs a metaphor of a stranger approaching him on the street and offering money. This analogy could imply that he perceives receiving payment from “*a random person*” as an unusual experience, one that might not fit into his usual way of making sense of the world. The metaphor may also indicate that this experience is difficult to articulate directly, as it could be interpreted as falling outside his or society’s normative expectations of hobbies.

It is noteworthy that, in this hypothetical scenario, Kevin imagines refusing payment, following it with the statement: “*the kind of person that I am”*. In many ways he seems to interpret his own identity in a way whereby rejecting financial compensation feels more aligned with his sense of self. Yet, Kevin acknowledges that he receives payment for every stream.

Despite finding these transactions awkward, Kevin simultaneously experiences them as affirming. There is a notable tension between these feelings in that, while he struggles with the idea of accepting money, he also makes sense of donations as a form of validation. Payment signifies investment and appreciation, legitimising his work in ways that words alone might not. This internal conflict highlights a paradox: he is uncomfortable with receiving money because of how he perceives himself, yet the act of being paid reinforces his value within the community and the perceived legitimacy of his labour. Stewart also expressed dilemmatic feelings in relation to streaming:

***Excerpt 10, Stewart:** When it comes to donations and whatnot, cause at the end of day, these are all people who I thought just find… like I’m just, I just want to be their friend, you know? Like I wanna spend time with these people. I don’t want to just like—I don’t want to take their money, you know? Like, just like it took me a while to realize that the dynamic is such that that’s expected and that’s OK.*

Stewart seems to experience an emotional conflict when it comes to receiving donations, describing his understanding of donations as rooted in a perceived contradiction between financial transactions and his understanding of friendship. For Stewart, streaming appears to be an extension of social connection, making payment feel misaligned with the nature of these relationships. His account suggests discomfort with the idea of taking money from his viewers, as it challenges his self-perception as a friend rather than an entertainer.

However, over time, Stewart appears to reinterpret donations differently and reconciles this expectation within the streaming context over time. His sense making shifts, and he interprets receiving donations as part of an established and acceptable dynamic. By acknowledging that donations are “*expected and that’s OK*”, Stewart signals a shift in his understanding, moving from seeing them as uncomfortable to potentially recognising them as expressions of support. This interpretative shift allows Stewart to redefine boundaries between friendship and financial reciprocity. Eventually, he comes to see donations as reciprocal gestures that align with social expectations related to his role. This allows him to reconcile receiving donations with his sense of connection to viewers, without fully resolving the perceived contradiction between friendship and monetised exchange.

## 4. Discussion

This study explored experiences of Twitch partners’ mental well-being, sense of community formation/maintenance, and their role in facilitating mental health discussions. Using IPA, we interpreted how participants experienced benefits and challenges to mental health, demonstrating that while streaming provides a space for self-expression, social connection, and emotional support, it introduces significant psychological and emotional burdens. Participants’ accounts reveal dilemmatic tensions. While experiencing streaming as an enjoyable, self-fulfilling escape from stressors, and a means of self-preservation, they describe it as emotional labour, involving community-building and a need to carry affective burdens of others. Moreover, participants’ discomfort with monetisation reflects how some conceptualisations of labour position psychological and emotional support as secondary and therefore lacking material value, creating tension between self-conception and the realities of financial compensation.

Specifically, participants described streaming as an avenue for decompression, self-care, and fulfilment; echoing extant data on how online gaming and streaming mechanise stress relief and emotional regulation (C. Y. Chen & Chang, 2019; Sjöblom & Hamari, 2017). However, while serving as a valuable outlet, we can extend prior knowledge by highlighting how participants faced substantial emotional labour from streaming, particularly from audience interactions during disclosures of personal and/or mental health struggles. The sense of community that streamers curated and maintained, while largely positive, also made them feel a misplaced—and often emotionally taxing—responsibility for their viewers’ well-being, leading to struggles in establishing boundaries. Such findings present similarities with previous studies on the emotional labour of digital content creators, where parasocial relationships and harassment contribute to psychological strain, and streaming is constructed as “play labour” (Catá, 2019; Speed et al., 2023; Woodcock & Johnson, 2019). Though we can extend this into the Twitch space, specifically, the generation of specific theoretical frameworks as to the motivators and facilitators of such experiences, and their individual weighting, is a logical next step.

We extend prior research by illustrating how streamers make sense of their emotional labour. While some participants conceptualised it as an extension of their communal role, others struggled with the weight of unsolicited mental health disclosures, experiencing guilt and pressure to respond appropriately. This suggests that while streaming can be emotionally fulfilling, it requires the navigation of complex ethical and emotional challenges, reinforcing the need for greater structural support for content creators; mirroring emerging data in Wallace et al. (2026).

Before extending this interpretation further, it is important to clarify that the broader concepts discussed below are used as theoretically informed extensions of participants’ accounts, rather than as direct empirical claims made by participants themselves. Read through a critical psychology lens, participants’ accounts may also be situated within broader structural conditions in which formal mental health provision is often limited and care is displaced into informal community spaces. In this context, for example, austerity-oriented policies contribute to insufficient healthcare and mental health provision, globally; perhaps also placing the onus of care provision onto members of the community while limiting their capacity to provide care, thereby resulting in diminished wellbeing (Rousaki et al., 2024). As a result, streamers are interpellated into providing emotional labour, which contributes to feelings of alienation as their emotions become commodified within a system driven by monetisation. This involves both a loss of control over their labour and its processes, as well as the transformation of emotions into consumable products, thereby commodifying the support as well as emotional labour they provide (Brook, 2009; Stulikova & Dawson, 2023).

In many ways, participant experiences reflect intense dilemmatic tensions. They construct streaming in a positive light, experiencing it as an escape from other stressors and as a form of self-preservation and describe it as a source of enjoyment and self-fulfilment. Simultaneously, they experience streaming as a dual form of labour: building a community and fostering emotional connectivity, whilst performing substantial affective work involving carrying the emotional burdens their viewers place upon them. In itself, this presents a conceptually new finding within this niche population. The need to set emotional and relational boundaries was highlighted, with streamers describing the challenge of maintaining a balance between engagement and self-protection when faced with negative interactions such as trolling and criticism. This emphasises the emotional toll of navigating scrutiny, with participants linking audience feedback to early experiences of validation and rejection. These findings mirror research on the vulnerability of digital creators to external validation and the psychological consequences of negative feedback (Kim & Kim, 2022; Robinson, 2019).

Moreover, we expand extant literature by showcasing strategies employed to mitigate emotional harm, such as strict moderation or disengaging from hostile interactions. Despite being somewhat effective, they do not necessarily represent empowerment. Instead, they embody the individualisation of responsibility, whereby the burden of managing harassment and emotional strain is placed on streamers rather than on platforms or communities. This reflects neoliberal governance; shifting accountability for systemic problems onto individuals, framing them as responsible for self-regulation and resilience in contexts shaped by broader structural inequities (Brown, 2015). As such, this finding highlights the increasing need for mental health resources for streamers as well as their audiences.

Importantly, this process should not be understood as gender-neutral. Although this study was not designed as a gender-based analysis, the forms of self-management, responsibilisation, described here are likely shaped by gendered norms, and therefore, future research should examine how gender shapes streamer identity, emotional labour, audience expectations, and the negotiation of care within monetised platform communities. For example, Anna’s account further suggests that emotional responsibility may be experienced in gendered ways; while this study cannot support a systematic gender analysis, her experience should not be treated as directly equivalent to those of male participants.

Financial implications were also discussed. Participants expressed ambivalence to monetary support from their audiences, initially perceiving it as misaligned with their identity and their sense-making of communal relationships. Over time, however, perspectives shifted, interpreting financial contributions as validation and appreciation. This resonates with literature on the affective economies of digital labour, where financial support often carries emotional meaning (Wolff & Shen, 2024). Streamers’ relationship with compensation is complex. On one hand, being paid for emotional labour feels at odds with self-conception, potentially subverting their identities as they attempt to resist the commodification of support they provide. On the other, monetisation-related discomfort may reflect broader conceptualisations that frame psychological and emotional support as secondary and therefore undeserving of material value. These conflicting experiences highlight what Stulikova and Dawson (2023) discuss as the double hermeneutic of emotional labour. Our findings explicate the emotional and identity-related tensions arising when streamers attempt to reconcile receiving financial incentives with their identity-related sense making; while financial compensation validated their efforts, it introduced dilemmas about the authenticity of their relationships with viewers. This highlights the precarious nature of digital labour, where emotional and financial stakes are intertwined.

### 4.1. Implications and Recommendations

Our findings have implications and recommendations for academia and policy; highlighting the importance of studying mental health impacts on both viewers and content creators. Though extant research has documented mental health benefits of online communities (Gandhi et al., 2021; Uttarapong et al., 2022), we highlight the need to consider the well-being of those facilitating, curating, and maintaining these spaces. Future research is therefore recommended to investigate long-term psychological effects of streaming, the efficacy of coping strategies, and how emotional and digital labour is conceptualised.

Our findings do not establish the efficacy of any specific policy or intervention. Rather, they identify areas that platforms, researchers, and policy stakeholders may wish to consider and evaluate empirically. These include clearer signposting to mental health resources, guidance on managing viewer disclosures, moderation support for harassment and trolling, and optional training around boundaries and emotionally charged interactions. Future research should examine whether such forms of support are acceptable, feasible, and effective for streamers across different platform contexts. Finally, we highlight broader societal implications of digital labour and affective economies. As streaming continues to grow as a profession, it is essential to consider ethical dimensions of emotional labour in online spaces and the responsibilities of both platforms and audiences in ensuring a healthier digital environment, especially in the context of such communities normalising financial expenditure in return for online interaction.

### 4.2. Limitations

Despite its novel findings, this study has limitations. First, while IPA provides rich, in-depth accounts of participant experiences within a niche demographic, the sample was small and predominantly male. Generalisation was not intended; however, the gender imbalance limits the extent to which we can examine how emotional labour, harassment, audience expectations, and boundary-setting may be gendered. Second, recruitment through snowball sampling may have introduced self-selection bias, as streamers who felt able or willing to discuss mental health and emotional labour may have been more likely to participate. Third, the sample consisted of Twitch Partners, who occupy a monetised position within the platform. Their experiences may therefore differ from those of smaller creators. Fourth, the cross-national nature of the sample may have shaped participants’ experiences in ways that could not be fully explored within the scope of this study. Fifth, the study focused only on streamers’ accounts. Future research should incorporate viewer and moderator perspectives to better understand how emotional support is negotiated across streaming communities. Finally, interviews were conducted between 2022 and 2023, and livestreaming and platform policies/monitisation as well as norms around mental health disclosure continue to evolve. The findings should therefore be read as situated within this specific technological and cultural moment. Moreover, as gender was not an explicit focus of the study and the sample included only one woman, we do not claim to offer a systematic gender analysis. Instead, our findings point to gender as a key analytic dimension for future research on streamer identity and platform-based emotional labour. Finally, it should be noted that one of the participants was interviewed over two days owing to weather conditions causing poor internet quality; theoretically impacting data quality.

### 4.3. Conclusions

This study contributes to a body of research on digital mental health by explicating the sense-making of streamers in relation to their well-being, their negative/positive experiences regarding community formation, and dilemmas they face when receiving donations. The findings illustrate the complex and conflicting ways in which streamers navigate the emotional demands of their role and experience balancing personal fulfilment with the psychological demands of content creation. As streaming platforms continue to evolve, it is crucial to consider how digital labour practices impact mental health and to develop sustainable support systems for those at the forefront of online community-building.

## Figures and Tables

**Table 1:** Thematic table with analytical focal points and assigned participants.

Superordinate Theme	Subtheme	Core Analytic Focus	Illustrative Participants
**1. Positive experiences of streaming**	**1.1 Streaming as a coping mechanism to psycho-social challenges**	Streaming was experienced as a source of decompression, escapism, self-care, fulfilment, and psychological relief from everyday stressors.	Jared, Peter, Stewart, Anna
	**1.2 The joy and responsibility of facilitating communities**	Participants made sense of streaming as a relational and community-building practice, involving connection, shared celebration, emotional support, and responsibility toward viewers.	Stewart, Jared, Anna, Peter
**2. Negative psycho-social sense-making in relation to streaming**	**2.1 Emotional labour and a sense of responsibility**	Streamers described the emotional burden of responding to viewer disclosures, trauma dumping, and expectations of informal support, while negotiating limits around their role.	Jared, Anna, James, Stewart, Peter
	**2.2 Managing negative feedback**	Participants described how trolling, criticism, rejection, and hostile audience interactions affected mood, self-worth, and boundary-setting practices.	Kevin, Anna, James, Peter, Stewart, Jared
**3. Emotional dilemmas regarding stream-related payments**	**3.1 Ambivalence around donations and subscriptions**	Monetary support was experienced as both validating and uncomfortable, creating tensions between friendship, authenticity, labour, financial reciprocity, and community belonging.	Kevin, Stewart, Jared, Peter, Anna, James

## Data Availability

Raw transcripts are not available; however, our interview schedule is available here: https://osf.io/hgxuz/?view_only=6eced043e2ea4eb59c3a5a6164292d27, accessed on 6 July 2026.
